# eMindfulness Therapy—A Study on Efficacy of Blood Pressure and Stress Control Using Mindful Meditation and Eating Apps among People with High Blood Pressure

**DOI:** 10.3390/medicines2040298

**Published:** 2015-10-16

**Authors:** Matthew Tedder, Lu Shi, Mei Si, Regina Franco, Liwei Chen

**Affiliations:** 1 Healthcare Genetics Program, School of Nursing, Clemson University, 500 Edwards Hall, Clemson, SC 29634, USA; E-Mail: mltedde@clemson.edu; 2 Department of Public Health Sciences, Clemson University, 500 Edwards Hall, Clemson, SC 29634, USA; E-Mail: liweic@clemson.edu; 3 Department of Cognitive Science, Rensselaer Polytechnic Institute, 110 8th Street, Troy, NY 12180, USA; E-Mail: sim@rpi.edu; 4 Center for Integrative Oncology & Survivorship, Greenville Health System, 900 W. Farris Road, Greenville, SC 29605, USA; E-Mail: rfranco@ghs.org

**Keywords:** mindfulness based stress reduction, intermittent fasting, blood pressure reduction, smartphone

## Abstract

**Background:** With the increasing availability of Smartphones and wearable tracking devices, it is now feasible and affordable to apply such mobile devices to delivering mindfulness-based stress reduction (MBSR) and intermittent fasting (IF) to lower blood pressure, as traditional MBSR and IF incur the burden of commuting to the intervention sites for the patients. Our study will develop and scientifically evaluate an MBSR app, an IF app and an MBSR + IF app in terms of their effectiveness for lowering blood pressure. We will further explore the possible interaction effect (synergistic effect) between MBSR and IF intervention: will improved mindfulness enhance patients’ adherence to the IF protocol? **Methods:** We will develop an MBSR app, an IF app, and an MBSR+IF app. We will then conduct an 8-week randomized controlled trial with a factorial design to evaluate the efficacy of these new apps, especially the interaction effect between MBSR and IF. Eligible individuals will be randomly assigned to Group 1 (MBSR app), Group 2 (IF app), Group 3 (MBSR + IF app) or Group 4 (usual care). **Discussion:** This will be the first attempt to explore the impact of mindfulness intervention on the adherence of a behavioral intervention. Nevertheless, our protocol is limited in that the effectiveness of intermittent fasting on lowering blood pressure has not been supported by large-sample randomized controlled trials. Thus if there is no significant effectiveness we cannot determine whether it is due to the intermittent fasting intervention itself or it is due to the limit of smartphone as a vehicle.

## 1. Introduction

Hypertension and prehypertension are a growing problem in the US with nearly 60 million people having a blood pressure (BP) in the prehypertensive range [1]. Pre-hypertension, though not identified as a disease, has far-reaching implications on cardiovascular risk [2,3]. While pharmacological treatments are available for pre-hypertension and hypertension, lifestyle interventions are still the preferred approach for BP management [4,5]. With the increasing availability of Smartphones and wearable tracking devices, it is now feasible and affordable to apply such mobile devices to delivering mindfulness-based stress reduction (MBSR) and intermittent fasting (IF) to lower blood pressure, as traditional MBSR and IF incur the burden of commuting to the intervention sites for the patients.

It was estimated in 2013 that the average U.S. consumer spent around 2 h and 7 min a day inside mobile applications [6]. This has led to a surge in the number of applications available to download in both the Apple App Store and Google Play systems, respectively [7]. With the increasing prevalence of smartphones and their associated web-based applications, a rise in the use of this technology to deliver mobile health interventions has also been seen [8,9]. These technologies allow users to overcome various limitations to traditional interventions such as time, resources, flexibility, availability, and accessibility [10]. They have the potential to increase cost-effectiveness by reducing the time input from a therapist or other healthcare professional, increase participation in therapeutic activities outside usual clinical hours of operation, and reduce transportation cost associated with attending a health program and commuting to meetings. 

One increasingly popular field in health-based mobile applications is smartphone-based mindfulness apps, as a recent study has identified over 500 applications focused on the concept of mindfulness [8]. Mindfulness is defined as the nonjudgmental awareness of experiences in the present moment [11]. Many programs have been developed over the years that seek to improve the trait of mindfulness, and one of the most notable mindfulness intervention protocols is mindfulness-based stress reduction (MBSR) [12]. MBSR programs have been shown to increase this present centered awareness [13,14] and attention [15,16,17]. Qualitative analyses have had similar results with participants commenting on improved awareness and concentration as well as less mind wandering [18]. Additionally, MBSR has been shown to have positive results in populations suffering from stress, eating disorders, depression, obesity, and attention control [19,20]. Studies utilizing traditional MBSR methods have shown significant reduction in systolic (SBP) and diastolic (DBP) blood pressure, compared to the control group [1,21,22,23,24,25]. Despite the number of mindfulness applications available and the amount of information supporting the use of traditional MBSR methods, less is known concerning the benefits of smartphone delivered MBSR interventions.

A 2013 study by Plaza *et al*. determined that despite the number of smartphone based mindfulness applications, there was very little information supporting their usefulness [9]. Since that time a few studies have been performed that seem to support the use of mindfulness based mobile applications. Chittaro and Vianello developed an application devoted to a technique in mindfulness practice known as thought distancing and compared it to two separate traditional methods of practicing this technique in individuals with little or no meditation experience [26]. It was reported that participants achieved a higher level of decentering (the goal of this particular technique) while using the mobile application compared to the traditional methods. It was also noted that participants rated the mobile application more pleasant as well as less difficult to use. A randomized controlled trial (RCT) found that participants who accessed a mindfulness application for 10 days reported a significant increase in positive affect and reduction in depressive symptoms while no affect was seen in the control group [27]. It should be noted no significant differences were seen with regards to satisfaction of life, negative affect, and flourishing measures in the experimental group; however, it was suggested the short duration of the trial could be an explanation for this finding. An RCT harnessing a 2-week online mindfulness intervention found significant differences in mindfulness, depression and anxiety, and perceived stress with no difference reported in the control group [28]. Other studies using smartphone based mindfulness apps have provided similar significant differences of within-group effects on measures of stress, heartbeats-per-minute, and depression for participants using the intervention; however, between group effects were not found [29,30]. However, the effect of an MBSR mobile application on the measure of BP has not been evaluated by randomized controlled trials, leaving an important research gap.

Similarly, other behavioral interventions shown to have positive effects on lowering BP in the traditional setting have also not been evaluated using a smartphone based application, such as intermittent fasting (IF). IF is a dietary approach for outcome improvement among chronic disease patients [31]. This regimen contains a “feast” period in which food intake is *ad libitum*, and a “fast” period when calorie intake is restricted. Many of the cardiovascular benefits of fasting were first recognized during times of religious fasting [32,33,34]. Several studies in rats have suggested IF can decrease BP along with other cardiovascular risk factors [5,35]. So far, many studies among humans have confirmed the cardiovascular benefits of various forms of fasting [36,37,38,39,40,41,42]. Two experiments with overweight and obese women have demonstrated the impact of IF on lowering blood pressure [43,44]. While the mechanism for BP lowering from fasting is thought to be partially due to weight loss, studies showing a decrease in BP without significant weight loss as well as in normal weight individuals suggests a more complex relationship [38,45]. Other mechanisms such as decreased sympathetic nervous system activity through decreased insulin secretion and increased insulin sensitivity as well as decreased age-related vascular changes have been proposed [46,47,48]. Despite the evidence of cardiovascular benefits such as lowering BP from IF, so far no studies have evaluated the effectiveness of a smartphone-based IF app. The lack of information available related to the BP lowering effect of smartphone-based IF and MBSR apps as well as the evidence supporting this effect in traditional methods necessitates research to fill in this gap. Also, the potential for an interaction effect between these two interventions resulting in a greater effect on blood pressure suggests there is merit in combining them to test for an interaction.

Various environmental cues affect peoples’ food intake volume, especially when the eater has not been mindful of what he or she has been eating [49]. Food over-estimation could come from environmental factors such as package size and labels, plate size, and meal size. Additionally, the person being in a mindless state could easily influence these cues leading to over-consumption. Mindfulness training thus might provide an effective way of reducing our mindless “choice” of food overconsumption by increasing awareness of present experiences and providing greater freedom from environmental cues [49]. The combination of a mindfulness intervention with an effective diet might lead to better compliance and greater outcomes [49] than a dietary intervention alone. This led to our hypothesis that the combination of IF and an MBSR program could be more effective at lowering BP in pre-hypertensive and hypertensive patients than either of the two interventions alone.

The selected IF regimen is expected to contribute to the potential decrease of mindless eating by removing distractions [49]. The IF approach focuses on frequency of meals instead of food choice. By combining the heightened sense of awareness found in mindfulness training with decreasing external distractions through IF, we hypothesize that there will be a decrease in mindless eating. This potential interaction between the interventions could achieve a greater reduction in BP. Finally, MBSR has been suggested to be effective in promoting improved coping responses [50,51,52], indicating it may promote increased adherence to dietary restrictions.

## 2. Experimental Section

Our specific hypotheses are:

- Hypothesis 1: The combination of MBSR and IF will have a significantly greater effect on lowering blood pressure than either stand-alone intervention and the control group.
- Hypothesis 2: The interaction effect of the MBSR and IF interventions on the outcome of BP will be mediated by the impact of MBSR on fasting adherence.

In Step I (six months), we will develop apps with self-administered trainings for MBSR, IF, and MBSR + IF. The new apps will have features to monitor the adherence to the treatment and track patients’ daily physical activities and energy intake. In Step II (4 months), we will conduct an 8-week randomized controlled trial (RCT) with a factorial design to evaluate the efficacy of these new apps, especially the interaction effect between the MBSR and IF. For participant recruitment, flyers will be distributed around the Clemson, Anderson, Seneca, and Greenville areas containing study information and requesting participation. Individuals will be asked to complete a screening survey to determine eligibility based on the inclusion-exclusion criteria described in Appendix. Individuals deemed eligible will then be randomly assigned to Group 1 (MBSR), Group 2 (IF), Group 3 (MBSR + IF) or Group 4 (usual care). Patients will be assessed for all measures at baseline. Subsequently, the primary outcome (BP) and secondary outcomes will be measured bi-weekly and post-intervention.

### 2.1. Study Population

The study population will include 1428 men and women aged 25–50 years, diagnosed as having prehypertension (SBP of 120–139 mm Hg or DBP of 80–89 mm Hg) or stage-1 hypertension (SBP 140–159 mm Hg or DBP 90–99 mm Hg), and not taking antihypertension medications. Sample size was calculated using a power of 0.8 and a α of 0.05 in the STATA package of sampsi. The effect size of systolic blood pressure outcome was reported by Palta *et al* [24], as systolic blood pressure is the essential outcome measure of our study. According to the power analysis, 357 participants will be randomized into each of the four groups as described above, meaning that we will recruit a total of 1428 patients.

### 2.2. Smartphone-based Applications

The MBSR application created will be adapted from Kabat-Zinn’s MBSR program [12]. The application will be designed as an 8-week intervention with weekly instruction lasting 2.5 h. The application will have pre-recorded meditation guides that teach participants mindfulness techniques: body scan, sitting meditation, and yoga [1]. The sitting meditation guides will allow participants access to techniques such as mindful breathing and thought distancing; Participation in these practices for at least 45 min a day for six days a week will be included in the app. Additionally, the application will assign homework such as logging of daily practice. 

An adherence feature will also be developed for the apps that provide study developers information on the number of times the application has been used as well as the length of time spent in the application doing guided meditation. Thus we obtain real-time data about the usage of these apps, one potential new metric of adherence in the age of eHealth. For each week these apps will ask the smartphone user a question about their adherence to the assigned intervention during the past week, and the users’ response will also become our measure of adherence. 

The created IF application will provide participants with a schedule to follow based on an IF regimen [38]. This will consist of a fast day on which food intake is restricted and a feast day on which food is consumed *ad libitum*. On fasting days participants will be taught to have high water intake and allowed to consume energy-free beverages, tea, coffee, and sugar-free gum. 

### 2.3. Control Group

The control group will be given information about the dietary approaches to stop hypertension (DASH) dietary regimen [53], which is a recommended dietary approach to achieve desired BP [53]. Brochures about DASH will be given to all participants. 

### 2.4. Outcomes

As for criteria for success, the following measures will be collected during the study. While BP improvement is considered the primary signal for the intervention success, improvement in other measures will signal success for our intervention as well. 

#### 2.4.1. Blood Pressure

BP will be taken in a noise-free environment while the patient’s arm is supported at heart level and both feet on the floor [1]. The measurement will be taken three times using a blood pressure cuff with 5 min between readings. The average of the three readings will be used as the recorded measurement.

#### 2.4.2. Mindfulness

Mindfulness will be measured using the five-facet mindfulness questionnaire (FFMQ) [54]. This is a self-report tool consisting of 39 items focused on changes in the tendency of participants to be mindful in their daily lives. Questions are answered using a 5-point Likert scale with responses ranging from 1-never or very rarely true to 5-very often or always true. This scale has been used in a similar study focused on technological delivery of an adapted MBSR program [28].

#### 2.4.3. Fasting Adherence

Adherence to the IF regimen will be monitored using a 24-h food recall. Participants will be asked to detail their food intake from the previous day and any fast day when a participant consumes food considered non-compliant.

#### 2.4.4. Stress

Stress will be evaluated using the 10-item perceived stress scale (PSS) developed by Cohen and Williamson (1988) [55]. This tool is used to measure how stressful an individual perceives their life with questions focused on how uncontrollable, overloaded, or unpredictable they view it. Responses are based on a 4-point Likert scale ranging from 0-never to 4-very often.

#### 2.4.5. Sleep Duration

Sleep duration per day will be collected using the wristband FitBit [56].

#### 2.4.6. Anthropometric Measures

Weight (kg), height (m), and waist circumference (cm) will also be measured at each visit. Weight will be measured to the nearest 0.25 kg using a balance beam scale with the participant having on no shoes and light clothing. Height will be measured to the nearest tenth of a meter using the height bar on the balance-beam scale. Waist circumference will be measured using a standard tape measure and placing it between the top of the participants’ hipbone and the bottom of their ribs while the breath is out. Measurements will be rounded to the nearest tenth of a centimeter.

#### 2.4.7. Physical Activity

Frequency and duration of walking, other moderate-intense activities, and vigorous-intense activities will be measured by the wristband Fitbit.

### 2.5. Analysis

We will use a mixed model analysis [57] to evaluate the effectiveness of our interventions to determine if our interventions prove effective in improving the above-mentioned outcome measures. A difference-of-difference approach will be used when every individual is set up as the fixed effect. Within this difference-of-difference framework, the interaction effect will be tested using the least-square mean function of SAS Proc Mixed procedure [58]. 

## 3. Results and Discussion

With the continued prevalence of prehypertension and hypertension in the US population, effective treatment methods are needed to counteract this growing problem. Additionally, the enormous cost, both direct and indirect, associated with this condition represent a barrier to effective management of BP [59]. This presents an opportunity for effective, low-cost interventions to play a role in ameliorating this issue. Due to decreased contact with healthcare professionals, increased participation in therapeutic activities outside the typical clinical setting, and reduced costs associated with attending a health program and commuting to meetings, healthcare interventions delivered by mobile devices represents a new avenue for patients.

The proposed study will lead to the development of novel applications for smartphones that aid in self-management of BP. This research takes advantage of the low-cost and convenient nature of mobile health interventions, thus having the potential to reach those who find it difficult to come to a health center regularly. In addition, the low-cost interventions should help reduce hypertension-related cost in the health care system.

Our proposed study is innovative in that it tests the effectiveness of two separate smartphone-based interventions, MBSR and IF, on the outcome of BP as well as other measures such as stress and mindfulness. The results will also provide information about the possible interaction effect of IF and MBSR applications on BP. Finally, the study is also innovative in that it test the effectiveness of the MBSR application in increasing adherence of the IF application.

## 4. Conclusions

The increased prevalence of smartphone use has provided unique opportunities to harness this technology to deliver mobile health interventions. This protocol will test the ability of mobile health interventions to deliver interventions aimed at decreasing prehypertension and hypertension. Results from this study will provide an early foundation for combining MBSR and IF interventions and the potential efficacy of this combinatorial method for decreasing BP. Additional research will be needed to build off these findings and to continue to seek low-cost ways of delivering interventions to manage blood pressure**.**

## Appendix

Inclusion and Exclusion Criteria	
Inclusion Criteria	Patients diagnosed as having prehypertension or stage-1 hypertension.
	Consented to participation in trial.
	Age: 25–50 years.
Exclusion Criteria	SBP above 159 mm Hg.
	DBP above 99 mm Hg.
	Severe Hypercholesterolemia (total cholesterol >8 mmol/L)—A condition characterized by elevated serum cholesterol levels.
	Previous experience with meditation practices—Any previous history of yoga and/or meditation classes or instruction.
	Pregnancy and Nursing—Currently pregnant or breast-feeding.
	Liver Disease—Any previous diagnosis of hepatitis, cirrhosis, alcoholic liver disease, or other condition leading to liver dysfunction.
	Kidney Disease—Any condition resulting in kidney damage or decreased kidney function for over 3 months.
	Blood pressure lowering medication—Any medication prescribed by a physician for the purpose of directly lowering blood pressure.
	Eating disorder—A diagnosis of bulimia, anorexia, or binge eating disorder
	Cachexia/malnutrition or BMI <20—A condition leading to loss of weight, weakness, muscle atrophy, and loss of appetite.
	Uncontrolled Hyperthyroidism—Diagnosed overactive thyroid without controlled management of thyroid hormone levels.
	Poor mental health or dementia—Diagnosed as having Alzheimer’s Disease, Huntington’s, Schizophrenia or other cause for cognitive impairment or major psychiatric disorder.
	Porphyria—The heme molecule of hemoglobin functions incorrectly.
	Hypoglycemia—Blood sugar (glucose) is abnormally low.
	Diabetes Mellitus—Any history or current diagnosis whether diet controlled or on oral agents or on insulin.
	Illiteracy or not able not able to speak or read English language
